# 57 Ocular tuberculosis: a differential for chronic panuveitis

**DOI:** 10.1093/rheumatology/keac496.053

**Published:** 2022-10-07

**Authors:** Monali Thakkar, Angela Migowa

**Affiliations:** Department of Paediatrics and Child Health Aga Khan University Medical College East Africa (Nairobi, Kenya)

## Abstract

**Background:**

*Mycobacterium tuberculosis* is an organism responsible for causing chronic granulomatous inflammation leading to active or latent tuberculosis (TB). Both pulmonary and extrapulmonary infections; including the skin, the eye, the cardiovascular, genitourinary and gastrointestinal systems can occur. Within the eye, it can cause global disease ranging from anterior or posterior uveitis to retinal TB. Within this abstract we demonstrate a case of chronic panuveitis with a diagnosis of ocular TB.

**Methodology:**

Retrospective chart review

**Results:**

We reviewed a 9-year-old- male in the rheumatology outpatient clinic who was on treatment for ocular TB with methotrexate 2.5 mg once a week, prednisone 5 mg once a day, maxitrol eye drops once a day and prednisolone eye drops twice a day.

He presented with a red, painless left eye with deteriorating vision 2 years ago. Functional enquiry was unremarkable. He was noted to have a fixed dilated left pupil at 3-4mm but other examinations were normal. Extensive investigations were carried out, including an Interferon-gamma release assay (IGRA)- which was positive. Toxoplasma antibody test, *Treponema pallidum* Haemagglutination test, and chest X-ray were all negative. He was then started on the above-mentioned therapies with marked improvement.

A decision on increasing the methotrexate dose if steroid tapering showed no improvement was undertaken and a plan on reassessing the patient with ANA, ACE and HLA B51 was made, to ensure complete workup.

**Discussion and conclusion:**

Intra or extra ocular TB (OTB) manifestations may result as a direct inoculation of the organism in ocular fluid or as a systemic immune response initiated by an infection from a distant site. There is a myriad of ocular manifestations of OTB which make forming a clinical diagnostic criterion difficult. The gold standard for diagnosis is an ocular fluid culture showing organisms but the yield may be low and the risk of damage to surrounding structures may be high. PCR tests done on the sample may yield clinical information but non ocular tests, such as the IGRA and tuberculin tests may also be employed. Therapy depends on active *vs* latent disease and mainly involves anti-inflammatory agents and anti-TBs.

Ocular TB may lead to sudden and irreversible blindness with high socioeconomic implications and hence, an index of suspicion in areas with a high disease burden should be kept as diagnosis poses a challenge.

